# No brain MRI abnormalities after mild-to-moderate COVID-19: an observational study

**DOI:** 10.1007/s00234-025-03586-1

**Published:** 2025-05-02

**Authors:** Lukas Haider, Paulus Rommer, Iscel Ahmet, Alexandra Dena, Alexander P. Thurnher, Emanuele Tomassino, Diana Bonderman, Florian Thalhammer, Stefan Seidel, Thomas Berger, Majda Thurnher

**Affiliations:** 1 Section of Neuroradiology and Musculoskeletal Radiology, Department of Biomedical Imaging and Image-Guided Therapy, Medical University of Vienna, Vienna, Austria; 2 NMR Research Unit, Queen Square Multiple Sclerosis Centre, University College London Institute of Neurology, London, UK; 3 Department of Neurology, Medical University of Vienna, Vienna, Austria; 4 Fifth Medical Department with Cardiology, Favoriten Clinic, Vienna, Austria; 5 Department of Diagnostic Neuroradiology, Hôpital Neurologique - Université Claude Bernard 1, Lyon, France; 6 Department of Internal Medicine, Division of Cardiology, Medical University of Vienna, Vienna, Austria; 7 Department of Urology, Medical University of Vienna, Vienna, Austria

**Keywords:** Brain, SARS-CoV-2, COVID-19, Perivascular spaces

## Abstract

**Purpose:**

To assess COVID-19-related morphological brain changes in individuals who recovered from mild-to-moderate COVID-19.

**Method:**

This prospective cohort study enrolled 112 consecutive individuals who recovered from mild-to-moderate COVID-19 and underwent an MRI of the brain between September 2020 and March 2022. MR exams were consistently obtained on a clinical 3T MR scanner in all study participants and 50 age-matched matched controls. The following clinical neuroradiological MR imaging findings were analyzed: post- and acute ischemic lesions, cortical signal alterations, microbleeds, perfusion abnormalities, cytotoxic lesions of the corpus callosum, and vascular abnormalities. Additionally, we manually quantified white matter lesion loads and the number of perivascular spaces and performed an automated brain volumetric analysis.

**Results:**

In 112 consecutive individuals the mean age was 45 years, female: male = 70:42, mean days at MRI after SARS CoV-2 infection: 228 (sd: 140), and hospitalized: non-hospitalized ratio = 30:82. *Using general linear regression models*,* adjusting for age and gender*, the frequency of white matter hyperintensities was not significantly different between subjects who recovered from COVID-19 and matched controls: 9.8 (sd: 17.3) vs. 7.6 (sd: 12.7), *p* = 0.590. Similarly, the number of enlarged perivascular spaces was not significantly different between the two groups: 62.7 (sd: 43.5) vs. 61.3 (sd: 47.2), *p* = 0.902. A subgroup analysis between those who were hospitalized in the course of the disease, in which no one required intensive care, and those who remained outpatients, also did not reveal any differences in MRI measures. We did not find evidence for perfusion-/diffusion abnormalities, (micro-)hemorrhages, or cortical abnormalities.

**Conclusions:**

In the present cohort, there was currently no evidence of COVID-19-related morphological brain changes in individuals who recovered from mild-to-moderate COVID-19.

## Introduction

Coronavirus Disease (COVID-19) is a pandemic infection caused by the severe acute respiratory syndrome coronavirus 2 (SARS-CoV-2). One of the first retrospective analyses of subjects with COVID-19 in the Wuhan area revealed neurological symptoms in 78 of 214 COVID-19 patients [1]. While the frequency and the nature of neuroradiological involvement in subsequent reports, by the time of writing around 115 studies, have substantially varied, brain and spinal cord lesions were consistently associated with severe COVID-19 [2]. Among others, the reported MRI findings include infarcts, vasculitis, (micro) hemorrhages, posterior reversible encephalopathy syndrome (PRES), cytotoxic lesions of the corpus callosum (CLOCC), laminar cortical lesions, leptomeningeal enhancement, encephalitis, ocular involvement, and others [3–9].

The large spectrum of MRI findings likely reflects that central nervous system (CNS) involvement in the course of this pandemic disease could be caused by multiple disease-related factors, such as primary neurotropic viral inflammation, secondary hyperinflammation, para-infectious immune-mediated disorders, and critical illness, but also, study quality, ethnic heterogeneity, different viral strains, and others [10–16]. While neurological involvement is present in mild-to-moderate COVID-19, neuroradiological imaging findings have, however, been reported primarly in cases with severe COVID-19 [2, 10]. While evidence supports brain atrophy in non-hospitalized COVID-19 cases [17], data on detailed clinical neuroradiological reading are limited. It thus remains uncertain whether mild-to-moderate COVID-19 is associated with morphological brain MRI abnormalities in a combined clinical neuroradiological expert- and quantitative morphometric assessment.

We, therefore, prospectively conducted brain MRI in 112 individuals, without prior neurological history, after COVID-19. Subjects were either non-hospitalized or did not require intensive care unit support in the course of their COVID-19-related hospitalization. Imaging findings were compared to age-matched controls.

## Materials and methods

### Study cohort and definitions

One hundred and twelve consecutive individuals were recruited from two institutions between September 2020 and March 2022. The inclusion criteria were: recovery from reverse-transcriptase–polymerase chain reaction (PCR) confirmed COVID-19; mild-to-moderate COVID-19 disease course; absence of known prior neurological disease; and no contraindications for an MR examination [18].

The assessment of subjective COVID-19-related symptoms, including ageusia or hypogeusia, anosmia or hyposmia, fever, fatigue, headache, cough, and dyspnoea was performed around 3 months after the MRI visit via telephone and medical chart review if available.

Additionally, 50 age- and sex-matched individuals, referred to our department for clinical neuroradiological work-up of non-focal, non-emergency neurological symptoms prior to the COVID-19 pandemic, were included as a clinical control cohort. These individuals were found to have no morphological brain alterations after extensive analysis and normal brain MRI results.

A governing institutional review board approved the study protocol. Informed consent was obtained from each enrolled participant via in-person consent written process.

### Image acquisition

MR exams were consistently obtained on a clinical 3T MR scanner (Siemens Vida) in all study participants. The MR protocol included a 3D fluid-attenuated inversion recovery (3D-FLAIR), 3D-T1-weighted, coronal T2-weighted, diffusion-weighted imaging (DWI), arterial spin labeling (ASL), 3D Time-of-flight MR angiography (TOF-MRA), and susceptibility-weighted MR imaging (SWI). Post-contrast studies were not obtained. The parameters for the SWI sequence were: field of view (FOV) 220 × 172 × 130 mm (APxRLxHF); voxel size (VS) 0.6 × 0.6 × 1.5 mm; reconstruction matrix (RM) 960; time of echo (TE)/ repetition time (TR) 7.12ms/31ms; and flip angle (FA) 17°, with no water or fat suppression, slice thickness 1.5 mm.

### Image analysis

In the first step, images were neuroradiologically screened for structural abnormalities by a trained reader. In the next step, all cases were, independently, assessed by a senior neuroradiologist with more than 20 years of experience. The findings of both reading sessions were combined in a final consensus session, as reported in this manuscript.

The following MR imaging characteristics/findings were analysed for the presence/absence of: (a) post-ischemic defects (3D-FLAIR, T2, 3D-T1), (b) acute ischemic lesions (DWI), (c) cortical alterations (3D-FLAIR, T2, and 3D-T1), (d) microbleeds (SWI), (e) brain perfusion abnormalities (ASL), (f) cytotoxic lesions of the corpus callosum (3D-FLAIR, T2, and 3D-T1), (g) vessel abnormalities (3D TOF-MRA).

### White matter lesion counts

White matter lesions, consistent with small-vessel disease imaging characteristics, were identified and counted on FLAIR-3D images in all subjects [19].

### Perivascular space analysis

Perivascular spaces (PVS) were quantified as reported previously [20, 21]. In short, the frequency of sharply delineated structures of cerebrospinal fluid (CSF) signal intensity following the course and morphology of perforating vessels were counted in the centrum semiovale, the basal ganglia, and the midbrain on T1-weighted images [19]. In the manuscript, we report the total number of PVS, summed over the three locations.

### Volumetric analysis

The volumetric analysis was performed using the FDA-cleared AI-Rad Companion (AIRC) brain morphometry tool. It works via a tissue-wise segmentation model based on T1-weighted MPRAGE images and was accessed through Siemens SyngoVia [20, 22].

### Statistical analysis

The statistical analysis was calculated in R studio. In the text, quantitative data are provided with mean and standard deviation (SD) or median with a 25–75% range, as appropriate. The significance levels for group differences in white-matter hyperintensities (WMH) and PVS between mild-to-moderate COVID-19 subjects and controls were calculated with general linear regression models, adjusting for age and gender. Further, group comparisons were tested with non-parametric rank sum tests. Linear correlation was assessed with Pearson correlation coefficients, which are provided with a 95% confidence interval. The effects of mild-to-moderate COVID-19, hospitalization, age and sex on brain volumetric estimates were assessed with linear regression analysis. Exact, two-sided, p-values are given up to 10^− 4^ and *p* < 0.05 was considered statistically significant in the text.

## Results

### General cohort and controls

In subjects post COVID-19 (*N* = 112), the average age at MRI acquisition was 45 years (sd: 15), female: male (70:40), and, in controls (*N* = 50), 44 years (sd: 19), female: male (28:22). The average time of MRI examination was 228 days (sd: 140) after the COVID-19 diagnosis. Subjects with COVID-19 were non-hospitalized (*N* = 82) or hospitalized (*N* = 30), but none of them required intensive care unit support. Hospitalized COVID-19 subjects were older, at 50 years of age (sd: 17) vs. 44 years of age (sd: 14), *p* = 0.038.

No study participant had brain MRI scans prior to this study.

### Clinical symptoms

Subjective COVID-19-related fever, ageusia, anosmia, fatigue, limb pain, coughing, and dyspnoea, were acquired via telephone assessment and retrospective chart review in a subset of 50 individuals. Individuals were asked to rate the subjective extent of these symptoms within their first week after the COVID-19 diagnosis as absent (0), mild (1), moderate (2), and severe (3). No data were available in the two cases. In the remaining 48 individuals, headache was reported by 25, fever by 23, ageusia by 23, anosmia by 23, fatigue by 23, limb pain by 27, coughing by 24, and dyspnoea by 16 subjects. Headache was rated in median: 1 (25–75% range: 0 to 2), fever in median: 0 (25–75% range: 0 to 1), ageusia in median: 0 (25–75% range: 0 to 2), anosmia in median: 0 (25–75% range: 0 to 2), fatigue in median: 1 (25–75% range: 0 to 2), limb pain in median: 0.5 (25–75% range: 0 to 2) coughing, in median: 0 (25–75% range: 0 to 1) and dyspnoea in median: 0 (25–75% range: 0 to 1). While we observed a trend for higher anosmia and ageusia ratings in female subjects, the association did not reach the level of statistical significance in a model adjusting for age (*p* = 0.17010; *p* = 0.191).

### Neuroradiological analysis

We did not observe signs indicative of recent or non-recent embolic, territorial, or global ischemic lesions. MR perfusion did not show local or global asymmetries and MR angiography depicted smooth vessel calibers without irregularities indicative of vasculitis or occlusions.

Parenchymal SWI susceptibilities, consistent with (micro-)hemorrhages, were absent in all individuals. Laminar cortical lesions, as reported previously, were not observed.

No signs of cytotoxic lesions of the corpus callosum were detected. The McDonald 2017 criteria for dissemination in space were met in a 32-year-old female. Upon further inquiry, she recalled clinical symptoms consistent with a clinically isolated syndrome one year prior to her SARS-COV2 infection. This subject was excluded from the sub-analysis on white-matter lesion frequencies.

### Quantitative analysis

**Fig. 1:**
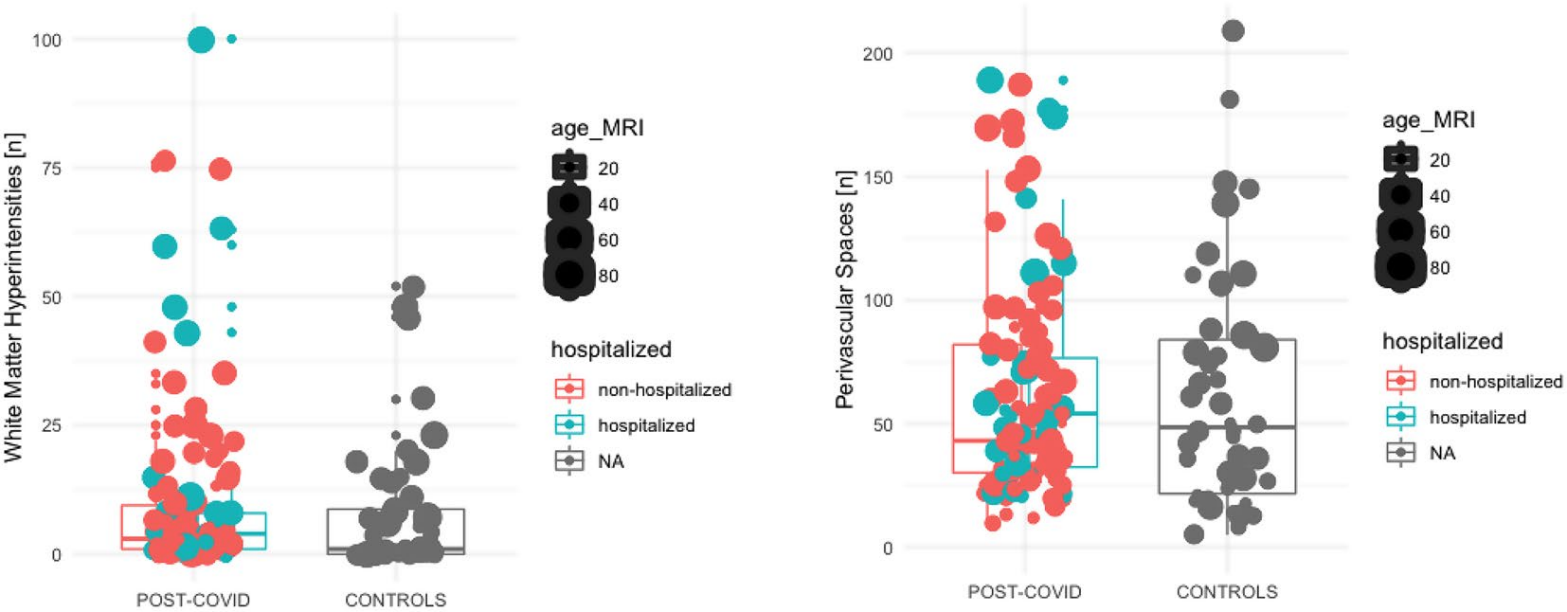
White-matter hyperintensities and perivascular spaces. Number of white-matter hyperintensities and perivascular spaces when comparing subjects with COVID-19 vs. controls. COVID-19 cases with hospitalization are blue, and those without are red. Data are provided with one dot per individual, and the group distribution is summarized with a median line and a 25–75% range box. The dot size indicates the age of the patient, and the color codes indicate hospitalization (yes/no)

The frequency of white-matter hyperintensities was not significantly different between subjects after mild-to-moderate COVID-19 and matched controls: 9.8 (sd: 17.3) vs. 7.6 (sd: 12.7), *p* = 0.590 (Fig. 1). In a sub-analysis within the COVID-19 cohort, comparing hospitalized vs. non-hospitalized subjects, similarly, no difference was found (*p* = 0.582) (Table 1).

**Table 1:** White matter hyperintensities and perivascular spaces

	Number of WMH: post-COVID vs. Controls			
**n_WMH**	**Post-COVID (** *N* ** = 112)**	**Controls (** *N* ** = 50)**	**Total (** *N* ** = 162)**	**p-value**
Mean (SD)	9.8 (17.3)	7.6 (12.7)	9.1 (16.0)	0.590
Range	0.0 to 100.0	0.0 to 52.0	0.0 to 100.0	
	**Number of PVS: post-COVID vs. Controls**			
**PVS**	**Post-COVID (** *N* ** = 112)**	**Controls (** *N* ** = 50)**	**Total (** *N* ** = 162)**	**p-value**
Mean (SD)	62.7 (43.5)	61.3 (47.2)	62.3 (44.5)	0.902
Range	10.0 to 189.0	5.0 to 209.0	5.0 to 209.0	
	**Number of WMH in post-COVID: non-hospitalized vs. hospitalized**			
**n_WMH**	**non-hospitalized (** *N* ** = 82)**	**hospitalized (** *N* ** = 30)**	**Total (** *N* ** = 112)**	**p-value**
Mean (SD)	8.3 (14.0)	13.7 (24.1)	9.8 (17.3)	0.582
Range	0.0 to 76.0	0.0 to 48.0	0.0 to 100.0	
	**Number of PVS in post-COVID: non-hospitalized vs. hospitalized**			
**PVS**	**non-hospitalized (** *N* ** = 82)**	**hospitalized (** *N* ** = 30)**	**Total (** *N* ** = 112)**	**p-value**
Mean (SD)	60.5 (41.5)	68.9 (48.7)	62.7 (43.5)	0.936
Range	10.0 to 187.0	21.0 to 189.0	10.0 to 189.0	

Number of white-matter hyperintensities and perivascular spaces when comparing subjects with COVID-19 vs. controls, and, within COVID-19, those who were hospitalized vs. those who were not. Group differences are adjusted for age and gender

**Abbreviations**: SD, standard deviation; WMH, white-matter hyperintensities; PVS, perivascular spaces

Enlarged perivascular spaces were noted in both groups and the total number of perivascular spaces was not significantly different between COVID-19 and controls: 62.7 (sd: 43.5) vs. 61.3 (sd: 47.2), *p* = 0.902 (Fig. 1). Similarly, when adjusting for age and gender, no difference was present within the COVID-19 cohort when comparing hospitalized vs. non-hospitalized subjects (60.5 (sd: 41.5) vs. 68.9 (sd: 48.7)). This was also consistent in a sub-analysis for all three regions analyzed: the centrum semiovale, the basal ganglia, and the midbrain (data not shown) (Table 1).

White-matter hyperintensities were correlated with perivascular spaces, *R* = 0.37 (95% confidence interval: 0.197 to 0.523, p-value < 0.001) and age in subjects post COVID-19, *R* = 0.433 (95% confidence interval: 0.266 to 0.571, p-value < 0.001).

Patient-reported, subjective COVID-19 related symptoms (including ageusia or hypogeusia, anosmia or hyposmia, fever, fatigue, headache, cough, and dyspnoea) which were acquired via telephone assessment or chart review, were not associated with quantitative MRI metrics.

Using linear regression analysis to explain total grey matter volume, age and sex emerged as significant predictors. Grey matter volume was found to decrease with subject age, with an estimated reduction of 2.44 ml per year (95% CI: -2.91 to -1.96, *p* < 0.001). Female participants exhibited significantly lower grey matter volumes compared to males, with an average difference of 61.13 ml (95% CI: -77.19 to -45.06, *p* < 0.001). However, no significant difference in grey matter volume was observed between post-COVID and controls (*p* = 0.390). Overall the model explained 52.5% of the variance in grey matter volume (R² = 0.525, adjusted R² = 0.516).

In a sub-analysis within the post-COVID group, regression analysis revealed similar effect sizes for age, 2.18 ml per year (95% CI: -2.78 to -1.58, *p* < 0.001) and female sex, 61.80 ml (95% CI: -80.44 to -43.16, *p* < 0.001). However hospitalization during acute COVID-19 was not associated with significant differences in grey matter volume (*p* = 0.293). The model accounted for 48.2% of the variance in GM volume (R² = 0.482, adjusted R² = 0.468).

## Discussion

While numerous MRI features have been reported in subjects after severe COVID-19, or atrophy pattern on T1-weighted images, the frequency of neuroradiological findings in mild-to-moderate COVID-19 is unknown. Our findings underscore the importance of addressing the real-world clinical scenarios in which individuals with mild-to-moderate COVID-19 are referred for brain MRI due to non-focal, non-emergency neurological symptoms such as persistent headaches, subjective cognitive difficulties, or prolonged anosmia. These symptoms, while not emergent, are clinically relevant and reflect the ongoing need for further investigation in post-COVID care. In the present study, in 112 subjects who were prospectively imaged after mild-to-moderate COVID-19, no evidence of brain involvement using structural and advanced MRI at 3T was found. While the absence of evidence is no evidence of absence, our findings indicate that, in mild-to-moderate COVID-19, after around 228 days (sd: 140), there is no morphological evidence to suggest CNS involvement in a sample size of 112. This observation has the following implications. First, our findings are reassuring that structural brain involvement, which could remain subclinical, was not missed on a large scale in mild-to-moderate COVID-19, while COVID-19 -related involvement, also in the mildest COVID-19 cases, is clinically suggested by ageusia and anosmia [23]. Similar to previous literature, we did not find evidence of gross morphological brainstem involvement, which could be hypothesized in the course of cranial nerve-related CNS entry via cranial nerve V/I [24, 25]. Second, in the approximately 115 publications on CNS involvement demonstrated on brain MRI in subjects with severe COVID-19, a broad spectrum of CNS pathology has emerged, including vascular/ischemic lesions, (micro-)hemorrhages, cytotoxic lesions of the corpus callosum, and posterior reversible encephalopathy [2, 13]. To some extent, these features might also be seen in critically ill individuals without COVID-19, and, to this end, our findings do not indicate a COVID-19-related pathology in mild-to-moderate COVID-19. However, the reported imaging findings in cases with severe COVID-19 are, by far, not fully explained by critical illness alone, and endothelial dysregulation, as well as irregularities in coagulation, have been identified as components of COVID-19-related brain involvement [26, 27]. The absence of CNS involvement in our cohort is in line with a retrospective study that showed a correlation between lung and brain involvement in hospitalized COVID-19 patients with acute neurologic manifestations [28]. Third, perivascular inflammation has repeatedly been shown in autopsy cases of COVID-19, and enlargement of the perivascular spaces occurs in the course of acute inflammation in other CNS conditions [13, 29]. However, we did not find evidence of higher perivascular space counts in our cohort, suggesting that perivascular inflammation in mild-to-moderate COVID-19 is either below the detection level of our analysis, or absent at the time of acquisition (at a mean of 228 days after a positive PCR). Our volumetric analysis reproduced known influence of age and sex on grey matter volume, but we did not detected effect of post-COVID status. Furthermore a sub-analysis within the post-COVID group showed no significant impact of hospitalization status on grey matter volume.”

While cognitive decline is now increasingly recognized as a sequale of COVID-19, the literature is largely focused on structural brain findings, with little to no data on structed clinical neuro-radiological assessment of extensive MRI protocols [30].

This study has several limitations. As the cohort was prospectively recruited, there is a wide age range and gender was homogeneously distributed, but the sample size is limited. Considering the previously reported frequencies of neuroradiological involvement, e.g., (micro-)hemorrhages in 6.9% (95% confidence interval, 4.9-8.9%), in a recent meta-analysis, it should, however, have been sufficiently powered to detect cases, if this feature was present at a comparable frequency in mild-to-moderate COVID-19 [31].

In the present analysis, an assessment of COVID-19-related subjective clinical symptoms was performed with phone calls and a retrospective chart review and was available only in 50 individuals. While acceptable test criteria can be achieved with telephone assessments in domains comparable to those investigated in the present manuscript, our data do not provide a detailed clinical assessment of symptoms significantly affecting functional outcomes after COVID-19 [32]. Further, we likely have a lower power to detect clinical symptoms and we are limited to the assessment of symptoms known to be associated with the disease. While symptom data were collected from a subset of participants, this was not the primary focus of our study nor directly related to our outcome measures. We acknowledge this as a limitation but believe that the absence of complete symptom data does not diminish the relevance or value of our findings on structural brain changes in this population.

The vaccination program started in Austria in February 2021 for healthcare professionals and individuals at risk. In 48/112 subjects, brain MRI was acquired prior to February 2021. While vaccination status, including type of vaccination or the time post vaccination, are unknown, a sub analysis between those that received their MRI before and February 2021 did not show any group differences, data not shown.

While COVID-19 infections were excluded by medical chard review in the control cohort, we cannot exclude sub-clinical or non-reported infections in both, controls and the COVID-19 group.

We cannot provide detailed information on SARS-COV2 mutation status in our patients.

The mean follow-up interval in this cross-sectional study, from clinical symptoms to brain MRI, was 228 days, we cannot exclude structural brain MRI findings prior to or after this time point, but if present they would have been reversible from an imaging point of view, and findings after this interval will be determined with a follow-up study.

As the absence of prior neurological morbidity and thus brain MRI, was an inclusion criterion, it is impossible to assess the temporal course of the encountered imaging findings. So, while we did not observe cross-sectional group differences between controls and individuals after mild-to-moderate COVID-19 in white matter lesions and enlarged perivascular spaces and other structural brain MRI findings, we cannot exclude faster accrual rates over time.

The set of imaging sequences we selected for this analysis was chosen based on previous literature in the field [2, 31]. However, we might have missed cranial nerve involvement or leptomeningeal enhancement (described in the literature after the study was initiated), which would have required the acquisition of post-contrast T1-weighted images.

The limitations of this study also include a lack of stratification of the severity of the cases, beyond the information of whether they had been hospitalized.

Due to the limited clinical information, we did not include comorbidities (smoking, hypertension, diabetes) in our analysis.

Last, but not least, the McDonald 2017 criteria for dissemination in space were fulfilled in a 32-year-old female that was excluded from the analysis [33]. While SARS-CoV-2-induced demyelination has been suggested, we hypothesize that our case rather resembled an incidental finding of multiple sclerosis, as symptoms (consistent with a clinically isolated syndrome) were, upon inquiry, recalled in the year prior to the SARS-CoV-2 infection [34, 35].

## Conclusions

Taken together in our cohort of 112 subjects, imaged around 228 days after mild-to-moderate COVID-19, we did not find evidence of structural brain abnormalities.
